# Environmental and Experimental Factors Affecting Efficacy Testing of Nonporous Plastic Antimicrobial Surfaces

**DOI:** 10.3390/mps1040036

**Published:** 2018-10-08

**Authors:** James Redfern, Jake Tucker, Lisa M. Simmons, Peter Askew, Ina Stephan, Joanna Verran

**Affiliations:** 1 School of Healthcare Science, Manchester Metropolitan University, Chester Street, Manchester M1 5GD, UK; J.Verran@mmu.ac.uk; 2 School of Engineering, Manchester Metropolitan University, Chester Street, Manchester M1 5GD, UK; jakestucker@hotmail.co.uk (J.T.); L.Simmons@mmu.ac.uk (L.M.S.); 3 IMSL, Pale Lane, Hartley Whitney, Hants RG27 8DH, UK; Peter.askew@imsl-uk.com; 4 BAM, Unter den Eichen 87, 12205 Berlin, Germany; Ina.Stephan@bam.de

**Keywords:** method development, standardisation, antimicrobial test, environmental conditions, hospital premises

## Abstract

Test methods for efficacy assessment of antimicrobial coatings are not modelled on a hospital environment, and instead use high humidity (>90%) high temperature (37 °C), and no airflow. Therefore, an inoculum will not dry, resulting in an antimicrobial surface exhibiting prolonged antimicrobial activity, as moisture is critical to activity. Liquids will dry quicker in a hospital ward, resulting in a reduced antimicrobial efficacy compared to the existing test, rendering the test results artificially favourable to the antimicrobial claim of the product. This study aimed to assess how hospital room environmental conditions can affect the drying time of an inoculum, and to use this data to inform test parameters for antimicrobial efficacy testing based on the hospital ward. The drying time of different droplet sizes, in a range of environmental conditions likely found in a hospital ward, were recorded (*n* = 630), and used to create a model to inform users of the experimental conditions required to provide a drying time similar to what can be expected in the hospital ward. Drying time data demonstrated significant (*p* < 0.05) variance when humidity, temperature, and airflow were assessed. A mathematical model was created to select environmental conditions for in vitro antimicrobial efficacy testing. Drying time in different environmental conditions demonstrates that experimental set-ups affect the amount of time an inoculum stays wet, which in turn may affect the efficacy of an antimicrobial surface. This should be an important consideration for hospitals and other potential users, whilst future tests predict efficacy in the intended end-use environment.

## 1. Introduction

There are an estimated 700,000 deaths recorded annually due to antimicrobial resistance (AMR), with this number expected to rise dramatically [1]. For many years, the front-line defence against bacterial infections was treatment with antibiotics [2]. Whilst antibiotics still remain an essential tool in tackling microbial infection, many scientists and engineers across academia and industry are looking for novel solutions for microbial control. Thus, there is an increasing focus on limiting the survival and growth of microorganisms in the hospital environment and preventing infection by cross-contamination. Advances in materials/coatings engineering have made antimicrobial materials/coatings (e.g., porous and nonporous surfaces, textiles) an attractive investment for infection control professionals. To illustrate interest in the field, as of May 2018, a Google Scholar search for “antimicrobial coatings” (AMCs) yielded 19,700 results. Further analysis of the literature shows that common antimicrobial coatings often contain nanomaterials with silver, chitosan, titanium, copper, zinc, and gold [3].

Antimicrobial coatings encompass a broad range of different modification-types including antiadhesive surfaces, contact-active surfaces, biocide-releasing surfaces, and modified topographies [4]. As mentioned above, many surface modifications use nanomaterials, which have unclear toxicological impact and lack of ‘safe-by-design’ procedures [3]. The variety of manufacturing techniques, coatings, additives, etc. make it difficult to create a catch-all list of criteria to confidently assert a surface as both safe and antimicrobial. 

Other issues concern the in situ life span and activation of an AMC. A recent systematic review of AMCs and their effectiveness in the healthcare environment found that whilst it is clear that copper surfaces harbour less microorganisms than noncopper surfaces, the literature is generally lacking in both quality and number of studies [5]. Copper is not the only AMC that has demonstrated antimicrobial efficacy, however there is a lack of studies demonstrating this in the healthcare setting. Also, because an AMC may require moisture and contact with a microorganism to be active (and therefore kill the microorganisms), consideration of cleaning protocols is also essential. Poor cleaning may fail to remove barriers between the microorganism and the antimicrobial activity (e.g., soiling/conditioning film). A recent discussion paper comprising the views of 75 contributors from academia and industry presented a unanimous demand for scientific guidelines to standardise cleaning of AMCs and also specified that prior to further implementation of AMCs, it is imperative that responsibility towards the continued effective use of AMCs in the hospital environment is specified [6]. 

However, ideally, before such implementation, the AMC must undergo effective and reproducible testing in vitro so that results from such testing are translatable into an environment more closely resembling the intended real-world application (e.g., hospital ward). Currently, harmonised test methods and general criteria to evaluate nonporous antimicrobial performance of surfaces are numerous but lacking in methodological diversity [7,8], with a number of test protocols based on the standard methods BS ISO 22196 and JIS Z 2801 [9,10]. In brief, this method requires the operator to inoculate the putative antimicrobial surface with a small known concentration and volume of *Escherichia coli* or *Staphylococcus aureus*, cover with a film, place in a petri dish, and incubate at a temperature of 35 ± 1 °C and a relative humidity (RH) of not less than 90% for 24 ± 1 h. Surfaces are then recovered into a validated neutraliser solution, which is then serially diluted and plated onto nutrient agar for CFU/mL^−1^ calculations. However, although this standard method provides a relatively simple approach to testing the efficacy of an antimicrobial material, it lacks substance in relation to real-world conditions, in which the use of the antimicrobial surface is intended. Whilst there have been many modifications to these standard test methods for antimicrobial coatings used in the literature, only one paper, to the author’s knowledge, specifically discusses methodological changes that would aim to present data more realistically to the real world [11].

For example, the UK Department for Health and Social Care guidance on healthcare premises recommends the humidity of a surgical ward to be 30–65% (no guidance is provided for general wards), a standard ward temperature band of 18 °C to 28 °C and an airflow of no more than 2 ms^−1^ at a vent face [12]. Since some AMCs require moisture to be antimicrobial, a dry surface will not present any antimicrobial efficacy, whilst a surface kept in an artificially high humidity (e.g., in an in vitro test chamber) may exhibit prolonged/increased antimicrobial activity. Thus, the drying time of liquids/moisture in real-world conditions need to be considered in the design of test methods. 

Changes in humidity and temperature will affect how long an inoculum will take to dry, and therefore when an experiment begins, and for how long an antimicrobial effect is likely to be sustained. Previous studies have highlighted how humidity has long been controlled in sealed environments using saturated salts [13,14], however, controlling the effect of saturated salts can prove difficult, with time taken to reach the intended humidity and time taken to re-establish a specific humidity if a test chamber is opened (to recover samples) providing confounding and often unspecified effects. Additionally, variables such as temperature, test chamber size, as well as volume, and surface area of saturated salts will also affect drying time.

This study provides an analysis of how environmental and experimental factors affect the behaviour of inoculum droplets on test surfaces, enabling methods based on BS ISO 22196:2011 to be developed that align more closely to environmental conditions likely to be found in a hospital ward.

## 2. Materials and Methods

### 2.1. Time Taken for Saturated Salt Solutions of Different Surface Areas to Reach a Specific Relative Humidity

Initial relative humidity measurements were made in test chamber 1 (TC1), a 15 L glass aquarium measuring 300 mm × 210 mm × 240 mm, with a detachable glass lid, which was removed to allow access to the test chamber. The lid was sealed to the main body of the aquarium with petroleum jelly. Inside the test chamber was a wire mesh platform for test samples (30 mm from the bottom of the aquarium), allowing space for Petri dishes underneath. The Petri dishes were used to contain a saturated salt solution of potassium carbonate (K_2_CO_3_) (selected because this salt solution should attain a RH% of 43%, a RH% within range of a surgical ward [13]) spread over one of four surface areas. For each surface area, 75 g of K_2_CO_3_ was mixed with 50 g of water (to create a slurry) and evenly spread over 64 cm^2^, 128 cm^2^, 192 cm^2^, or 256 cm^2^. The saturated salts were placed at the bottom of TC1. Once the lid was sealed, change in RH% was captured by a data logger inside the chamber at intervals of five minutes. The starting RH% was between 23% and 24.6%. To assess the time taken for the RH% to drop from the target RH% back to the starting RH%, the data logger continued to record RH% change once the lid of the chamber was removed. Due to the faster change in RH% (equilibrating to the outside environment), data were logged every 20 s over 260 s.

### 2.2. Effect of Environmental and Experimental Conditions on the Drying Time of Different Sized Droplets of Water

**Figure 1:**
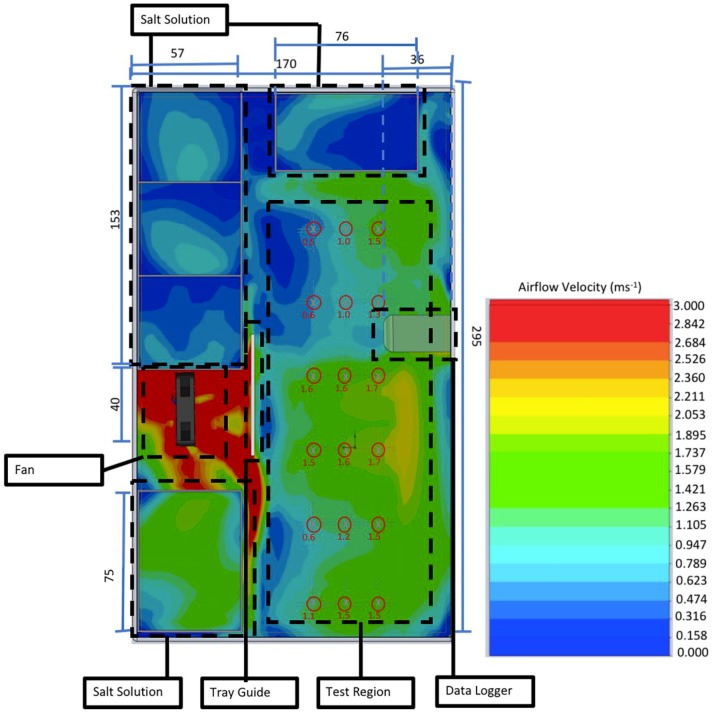
Schematic of test chamber 2 with a cut point of 1 mm above sample plane. All measurements on the outside of the box are in mm. Each area of the chamber is highlighted with a dotted line. Eighteen inoculum droplets can be placed on nonporous surface samples and placed within the test region. Numbers underneath the sample locations indicate airspeed (ms^−1^). Colour overlay represents airflow (ms^−1^), with colours corresponding to the key to the right.

Further development resulted in test chamber 2 (TC2—Figure 1), a polypropylene box (sealable with clips—Really Useful Boxes, Castleford) measuring 171 mm × 296 mm × 51 mm. The box contained a 40 mm × 40 mm × 10 mm 3D-printed cooling fan with a face airflow of 3 m/s at 10 V. The airflow within the box at 1 mm above the sample surface was modelled using Solidworks Computational Fluid Dynamic (CFD) analysis. Temperature was controlled with a thermostat (Vivosun, Shanghai, China) inside the test chamber. This informed a propagation heat mat (PVC, 18 W, 220 V) which was placed outside and beneath the box. Relative humidity and temperature were recorded using a HD500 sensor and datalogger (Extech Instruments) located inside the chamber. Five open-topped boxes for saturated salt solutions were located around the edge of the test chamber presenting a combined surface area of 164 cm^2^. To allow the operator to remove samples without opening the top of the test chamber (thereby undesirably equilibrating the RH of the test chamber to that of the outside environment), a sliding caddy was built into the side of the test chamber. The caddy allowed for the installation of six test samples measuring 75 mm × 25 mm × 2 mm, each of which could accommodate up to three droplets of liquid inoculum.

Environmental conditions inside TC2 were identified in advance and the box was left for 1 h for the interior to stabilise to the intended RH% and temperature. The salts used to create environments with a range of humidities included magnesium nitrate (Fisher Scientific, Loughborough, UK), lithium chloride (Fisher Scientific), and sodium chloride (Fisher Scientific). These were placed in the chamber using a range of different weights and water mixtures over different surface areas (Table 1). The heat mat was set at temperatures between 24 °C and 30 °C and the fan was operated at 10 V or 8 V to achieve different airflow speeds (Table 1). Temperature and RH% were recorded every 60 s using a data logger.

Six polypropylene test samples, measuring 75 mm × 25 mm × 2 mm, were loaded into the sliding caddy and each was inoculated with 18 droplets of 2 μL, 5 μL, 10 μL or 20 μL of distilled water. The caddy and samples were carefully loaded into the box, marking the start of the experiment. Each water droplet was observed at intervals of 120 s, with a droplet being considered ‘dry’ when no liquid was visible to the naked eye at a distance of 300 mm.

### 2.3. Linear Regression Mathematical Model

All drying time data collected in the previous experiment (630 data points) were used to create a linear regression model using MATLAB (V 2016b for Mac, Mathworks, Natick, MA, USA) to enable the development of a model that would predict drying the time of any size of water droplet when RH%, airflow, and temperature conditions are specified in advance by the user. 

### 2.4. Statistical Analysis

Statistical analysis was carried out using Graphpad Prism v7 (La Jolla, CA, USA). A significance cut off of α = 0.05 was used in all tests. Data on the difference between drying time and the range of temperature, RH%, and airflow were analysed using the Kruskal–Wallis test followed by the Dunn’s multiple comparisons test. 

## 3. Results

### 3.1. Time Taken for Saturated Salt Solutions of Different Surface Areas to Reach a Target Relative Humidity

**Figure 2:**
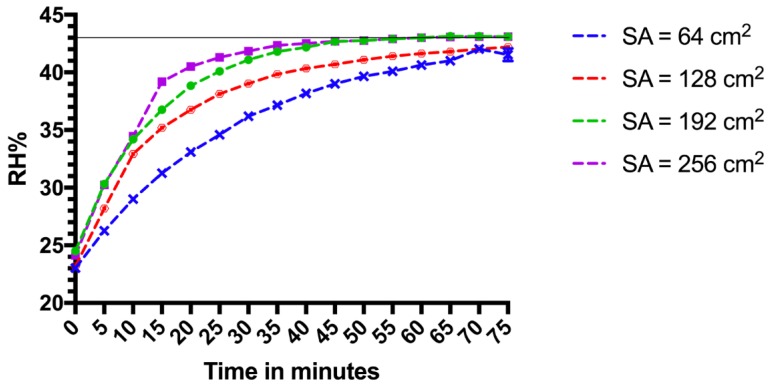
Time taken for relative humidity (RH)% to reach target of 43% (indicated by solid horizontal line). Each surface area contained 75 g of K_2_CO_3_ mixed with 50 g of water (to create a slurry) which was evenly spread over either 64 cm^2^, 128 cm^2^, 192 cm^2^ or 256 cm^2^. Data points were taken every five minutes. Each surface area was tested three times. SA = surface area of saturated salts.

**Figure 3:**
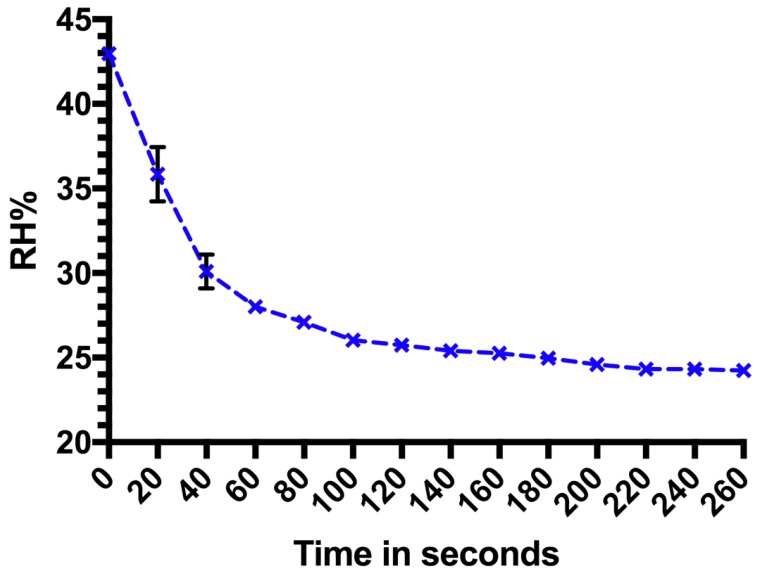
Time taken for RH% to revert from the RH% achieved using K_2_CO_3_ to the initial RH% after removing the lid from the test chamber after the experiments with 256 cm^2^ of saturated salts. Data points are pooled from three repeats and error bars (where visible) represent standard deviation.

The target RH% was 43%. When the saturated solution was spread over 64 cm^2^ and 128 cm^2^, the change of RH% took 70 min to come within a 1% margin of the target, but never attained 43%. When the saturated salt was spread over 192 cm^2^, it took 40 min to achieve a RH% within a 1% margin and 60 min to achieve 43%, whilst when spread over 256 cm^2^, the change in RH% took the same amount of time to achieve 43% but took less time (35 min) to get within a 1% margin (Figure 2). Thus, an increase in surface area increases the speed in RH% attainment. Time taken for the RH% to return to ambient conditions after the lid was removed (a drop of ≈19 RH%) was more rapid (Figure 3).

### 3.2. Effect of Environmental and Experimental Conditions on the Drying Time of Different Sized Droplets of Water

**Figure 4:**
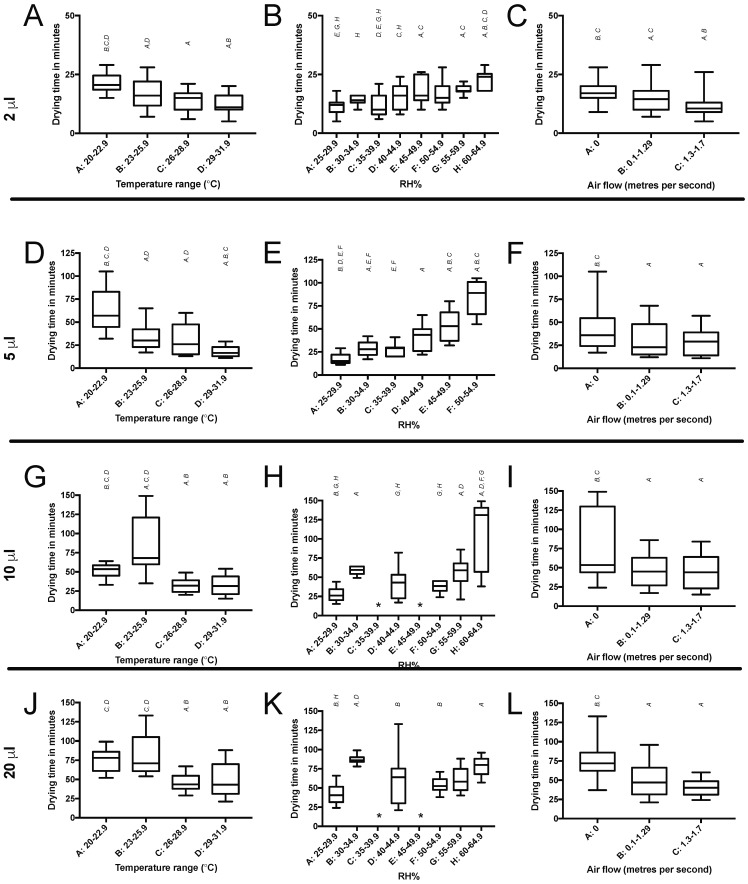
Drying time of either 2 µL (**A**–**C**), 5 µL (**D**–**F**), 10 µL (**G**–**I**) or 20 µL (**J**–**L**) droplets of water on plastic (polypropylene). The first graph in each row represents drying time related to temperature (°C). The second graph in each row represents drying time related to RH%. The third graph in each row represents drying time related to airflow (m/s). Vertical letters denote with which columns a statistical significance is shared (*p* < 0.05). Asterix denotes a category with zero data.

Drying times varied between 5 and 149 min depending on initial droplet volume and temperature, RH%, and airflow (Figure 4). The category with the lowest temperature (20–22.9 °C) produced a significantly (*p* < 0.05) longer drying time on average compared to all other (higher) temperatures for droplet sizes of 2 μL, 5 μL and 10 μL. The highest humidity category (60–64.9 RH%) extended drying time (*p* < 0.005) compared to the lowest. The time taken for droplets of all sizes to dry was significantly (*p* < 0.05) longer at 0 m/s airflow, compared to either 0.1–1.29 m/s or 1.3–1.7 m/s. 

### 3.3. Linear Regression Mathematical Model

**Figure 5:**
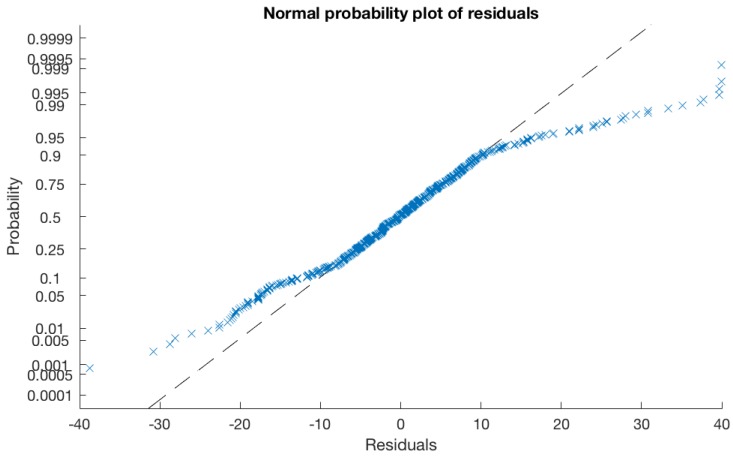
Normal probability plot of residuals illustrating a linear relationship and reinforcing the normal distribution assumption.

All data points were imported into MATLAB to generate a linear regression model (Figure 5 and Equation (1)). The model represented an R^2^ value of 0.872 (R = 0.934) with root mean square error of 10.4. This equation enables the estimation of drying time based on user-defined RH%, temperature, and air flow variables, or, if the user has a predetermined drying time they wish to achieve, the model can provide environmental parameters which will achieve the drying time. For an example of a user-journey utilising this model, see Table 2.

Equation (1): Equation for a linear regression model generated by the drying time of different sized droplets on a plastic surface in different environmental conditions (Figure 4):

*Y* ~ 73.003 − 107.99*X*_1_ − 4.6881*X*_2_ − 87.606*X*_3_ + 3.2635*X*_4_ + 101.25*X*_5_ + 6.2439*X*_6_ + 0.94088*X*_7_ + 4.1268*X*_8_ + 2.317*X*_1_*X*_7_ + 0.025487*X*_2_*X*_7_ − 1.2568*X*_3_*X*_4_ + 3.2719*X*_3_*X*_6_ − 2.4234*X*_5_*X*_8_, (1)

where: *Y* = drying time (m), *X*_1_ = average temperature (°C), *X*_2_ = average relative humidity (%), *X*_3_ = surface airflow (m/s), *X*_4_ = sample size (µL), *X*_5_ = minimum temperature (°C), *X*_6_ = maximum temperature (°C), *X*_7_ = minimum relative humidity (%), *X*_8_ = maximum relative humidity (%)

## 4. Discussion

The aim of this study was to assess whether variation in environmental conditions (compared to those found in the typical lab-condition AMC efficacy tests) changes the drying time of an inoculum, and thereby affects the data regarding antimicrobial efficacy. Temperature and airflow extended the drying time for droplets in which conditions matched those commonly found in a hospital ward (20–22.9 °C and of any airflow above zero m/s) when compared to those found in the current antimicrobial efficacy test method BS ISO 22196. Therefore, an antimicrobial coating undergoing efficacy testing using the BS ISO 22196 test conditions (35 ± 1 °C and a RH of not less than 90%) is likely to demonstrate a slower drying of test inoculum and enhanced antimicrobial effect, compared to what might be expected in a hospital ward. Similarly, a more humid environment increased the time taken for an inoculum to dry. Whilst the humidity conditions tested in these experiments are generally within the accepted range of RH% for an indoor environment, the current test conditions described in BS ISO 22196 are more humid, thus drying time is likely to be longer than the times described above. Thus, if the antimicrobial action of an AMC is triggered or enhanced by the prolonged presence of moisture, BS ISO 22196 is likely to provide a nonrealistic level of efficacy for an intended use in the hospital ward.

A proposed solution to this issue would be to take measurements of the drying time of a known inoculum volume in the proposed end-use environment, and use a mathematical model, such as that proposed here, to enable specification of the environmental test conditions needed to achieve a drying time in vitro that is similar to that expected in situ. For example, using formula 1, if an operator knew the droplet volume and target drying (as would be expected in situ), these data can be input into the model, along with a RH% measurement that is achievable in the test set-up, and the model will specify a temperature in which the experiment should be incubated to enable the target drying time to be achieved (Table 2). 

Currently, techniques used to test the efficacy of AMC are primarily designed with the operator in mind. Whilst this is understandable, it is important that environmental variables are kept as close to the intended end-use as possible, and that variation whilst testing is kept to a minimum. For example, removing the lid of a container to take out a sample at a specified time point will create a change in the RH%, which is likely to be unique to the given set up—reducing the reproducibility between different laboratories. Engineering solutions, which reduce these environmental shifts, such as the sliding draw described in TC2, should be considered as fundamental.

Whilst these data provide an initial insight into the variation between AMC efficacy test methods and the real-world conditions in which AMCs are expected to be functional, further work is needed to characterise the relationship between drying time and other surface types (e.g., polyethylene, HTPE), and in particular, characteristics such as hydrophobicity, wettability, and the effect of microorganisms inside the inoculum. In addition, understanding the relationship between drying time and textiles, in relation to AMC efficacy testing, is needed. Whilst many of the current standards and test methods utilised for efficacy testing of AMCs have a microbe-centric approach (with incubation temperature and humidity idealised for microbial survival) and include classic microbiology techniques, the system described in this paper provides a starting point for the inclusion of electrical/mechanical engineering, computer sciences, and other analytical techniques. The present system should help to create a more reproducible and reliable system for testing surfaces in laboratories across the globe unaffected by ambient climate and other environmental conditions.

## 5. Conclusions

This analysis of water droplet drying time in different environmental conditions demonstrates that experimental set-ups affect the amount of time an inoculum stays wet, which in turn may affect the efficacy of an antimicrobial surface. We suggest that efficacy tests should consider an inoculum drying time which is as close to the expected outcome in the intended end-use environment (e.g., hospital ward), which can be calculated using a model similar to that presented in this paper. 

## Figures and Tables

**Table 1:** Experimental and environmental conditions tested in test chamber 2 which were used to generate the linear regression model.

Salt (Target RH%)	Salt: Water Ratio (g)	Surface Area of Saturated Salt Container	Airflow (m/s)	Temperature of Heat Mat (°C)
Magnesium nitrate (53%)	4.5:1	29 cm^2^	0.5–1.9	28
	25:5	58 cm^2^	0	30
	25.8:7.5			
	51:7.5	87 cm^2^	0.4–1.5	30
	64.5:18.75	164 cm^2^	0–1.7	
Lithium chloride (11%)	12.3:7.5	87 cm^2^	0	24
	59.5:22.5	164 cm^2^	0–1.7	22, 26, 30
Sodium chloride (75%)	64.5:18.75	164 cm^2^	0.5–1.7	28
No salt/room conditions (35%)	n/a	n/a	0–1.7	26, 30

RH%: percentage of relative humidity.

**Table 2:** Example user-journey whereby the user utilises the model to determine an experimental set-up which will provide a drying time similar to what may be expected in the intended end-use environment.

Journey	Example Scenario
Determine drying time and environmental parameters at the intended end-use environment.	The drying time of a 20 μL inoculation into an antimicrobial plastic is known to be 200 min in the intended end-use environment (e.g., hospital ward).
Determine which environmental variables can be altered and which cannot in the laboratory undertaking the efficacy testing.	The user who is undertaking antimicrobial efficacy testing of the antimicrobial plastic is limited by the laboratory set-up, and cannot change the RH% of the test chamber.
The known information relating to expected drying time, average humidity, and sample size can be input into the model. The model will calculate a temperature value, which, if the test chamber is set to, should mimic the intended drying time.	The user sets the parameters of the model to an expected drying time of a 20 μL inoculum to 200 min in an environment of 55 RH%, which provides a temperature value of 12.75 °C.
This allows the user to undertake antimicrobial efficacy testing in conditions more realistic to the intended use.	The user sets the test chamber to a temperature of 12.75 °C to achieve the same drying time expected in the end-use environment.
